# A xandarellid artiopodan from Morocco – a middle Cambrian link between soft-bodied euarthropod communities in North Africa and South China

**DOI:** 10.1038/srep42616

**Published:** 2017-02-17

**Authors:** Javier Ortega-Hernández, Abdelfattah Azizi, Thomas W. Hearing, Thomas H. P. Harvey, Gregory D. Edgecombe, Ahmid Hafid, Khadija El Hariri

**Affiliations:** 1 Department of Zoology, University of Cambridge, Downing Street, Cambridge CB2 3EJ, UK; 2 Département Sciences de la Terre, Faculté des Sciences et Techniques-Guéliz, Université Cadi Ayyad, Avenue Abdelkrim el Khattabi BP 549, 40000 Marrakech, Morocco; 3 Department of Geology, University of Leicester, University Road, Leicester LE1 7RH, UK; 4 British Geological Survey, Keyworth, NG12 5GG, UK; 5 Department of Earth Sciences, The Natural History Museum, Cromwell Road, London SW7 5BD, UK

## Abstract

Xandarellida is a well-defined clade of Lower Palaeozoic non-biomineralized artiopodans that is exclusively known from the early Cambrian (Stage 3) Chengjiang biota of South China. Here we describe a new member of this group, *Xandarella mauretanica* sp. nov., from the middle Cambrian (Stage 5) Tatelt Formation of Morocco, making this the first non-trilobite Cambrian euarthropod known from North Africa. *X. mauretanica* sp. nov. represents the youngest occurrence of Xandarellida – extending its stratigraphic range by approximately 10 million years – and expands the palaeobiogeographic distribution of the group to the high southern palaeolatitudes of West Gondwana. The new species provides insights into the lightly sclerotized ventral anatomy of Xandarellida, and offers stratigraphically older evidence for a palaeobiogeographic connection between Burgess Shale-type euarthropod communities in North Africa and South China, relative to the (Tremadocian) Fezouata biota.

The Xandarellida Chen *et al*.^1^ (*sensu*^2^,^3^) are an enigmatic group of non-biomineralized artiopodan euarthropods whose distinctive features include the possession of stalked ventral eyes, a posterior extension of the cephalon covering the anterior trunk tergites, and the occurrence of dorsoventral segmental mismatch on the trunk^1^,^2^,^3^,^4^,^5^,^6^,^7^. Xandarellida consists of three taxa that are exclusively known from the Chengjiang Konservat-Lagerstätte (Cambrian Stage 3) in South China, namely *Xandarella spectaculum*^4^, *Cindarella eucalla*^1^, and *Luohuilinella rarus*^6^ (Fig. 1).

Within the diverse Palaeozoic clade Artiopoda Hou and Bergström^2^, xandarellids have been regarded as members of a more inclusive group known as the Petalopleura Hou and Bergström^2^ (Table 1), which also includes the lower Cambrian forms *Sinoburius lunaris*^4^ from Chengjiang, and (potentially) *Phytophilaspis pergamena*^8^ from the (Stage 4) Sinsk Formation in Siberia^9^,^10^. Unlike other monophyletic groups in Artiopoda, a clade that includes trilobites as its most familiar members (Fig. 1), the spatial distribution and temporal occurrence of xandarellids suggests a high degree of endemicity. Indeed, the group is conspicuously absent from Laurentia (North America) despite the intense study of numerous Cambrian Lagerstätten in this region^11^. Here, we describe an artiopodan interpreted as a xandarellid with appendicular preservation from the Cambrian (lowermost Stage 5) of the western High Atlas in Morocco. The new taxon represents the youngest stratigraphic occurrence of Petalopleura, the first palaeobiogeographic record of Xandarellida outside South China, and clarifies the organization of the lightly sclerotized ventral morphology in this poorly known group of non-biomineralized euarthropods.

## Geographic and Geological Setting

**Figure 2:**
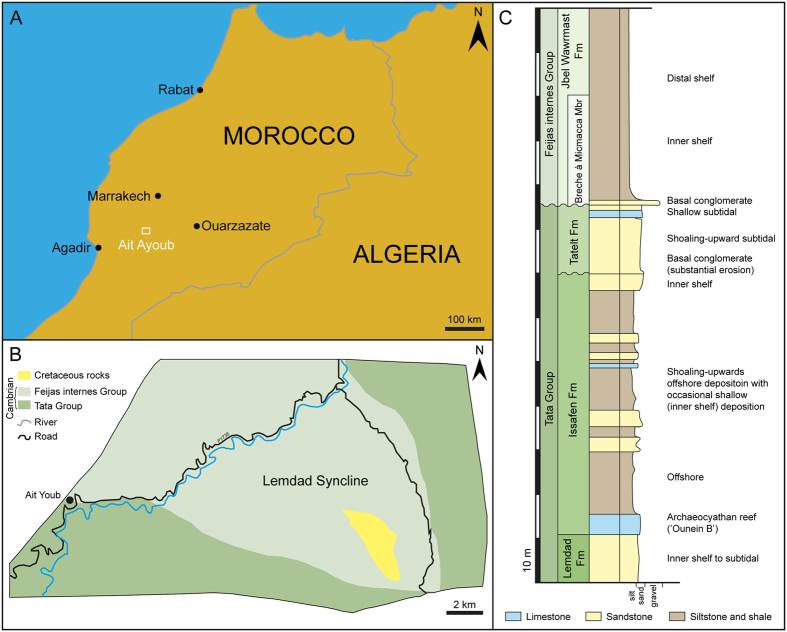
Geological setting of *X. mauretanica*. (**A**) Map of the locality within Morocco. (**B**) Geological sketch map of the Lemdad Syncline area. Redrawn, adapted and simplified from Geyer and Landing^15^. (**C**) Generalised sedimentary log through the lower – middle Cambrian (Tata – Feijas internes Group) transition in the eastern Lemdad Syncline; compiled from data in Geyer and Landing^14^,^15^. Maps (**A**,**B**) drafted by T.W.H. using Adobe Illustrator CC 2015.3 (http://www.adobe.com/uk/products/illustrator.html).

The Tatelt Formation (also referred to as the ‘Asrir’ Formation^12^) is exposed in the High Atlas Mountains of Morocco in the Lemdad Syncline (Fig. 2) and further south in the Anti-Atlas range. This unit is part of the Early Palaeozoic cover sequence deposited onto basement rocks of the Pan-African Orogen on the margin of West Gondwana^13^,^14^,^15^. The Tatelt Formation thickens southwards, from ca. 13–18 m in the Lemdad Syncline to ca. 55 m in exposures in the Anti-Atlas Mountains, with a concomitant transition from proximal to more distal facies^15^,^16^. The more proximal, High Atlas, succession is dominated by fine- to coarse-grained sandstones with intercalated grey-green tuff and ash beds, but also includes shale and conglomeratic layers^14^,^15^,^16^. The upper part of the Tatelt Formation in the Lemdad Syncline includes bidirectional trough cross-stratified layers and is interpreted as being deposited in a near-shore subtidal environment, with occasional intervals of deeper, or more quiescent, deposition^14^,^15^.

**Figure 3:**
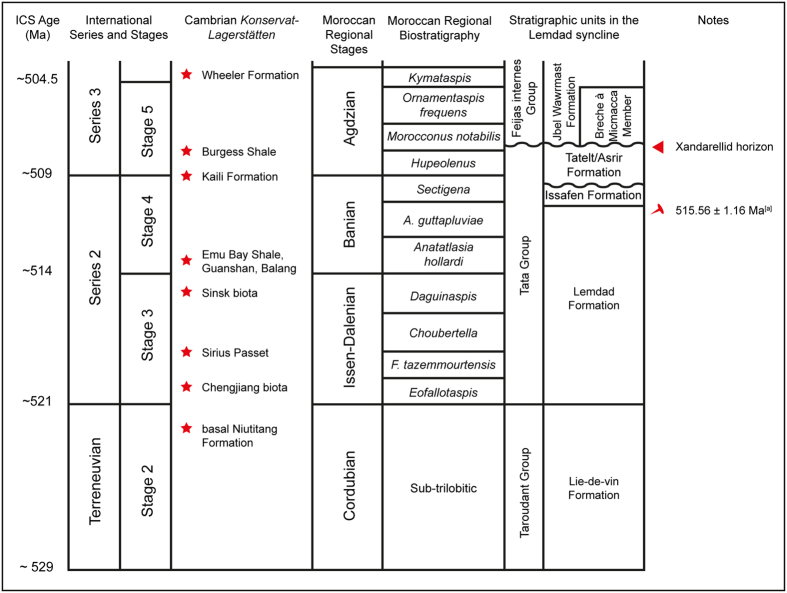
Generalised stratigraphy of the early - middle Cambrian transition in the Lemdad Syncline area, High Atlas Mountains, Morocco. Strata in the Lemdad Syncline correlated to the international geological timescale using the Moroccan regional biostratigraphic framework and shown alongside the relative ages of other Cambrian Lagerstätte with Burgess Shale-type faunas. Compiled from Geyer and Landing^15^, Geyer and Vincent^18^, Geyer and Malinky^19^, and Van Roy *et al*.^54^. ^[a]^Radiometric age for the upper Antatlasia guttapluviae Zone in the Lemdad Syncline recalculated^21^ after 517 ± 1.5 Ma^22^; note the discrepancy between this age and the international age (ca. 510 Ma) of Cambrian Series 2 Stage 4 to which the *A. guttapluviae* Zone has been biostratigraphically correlated^15^,^17^,^19^. Correlation of early and middle Cambrian strata in Morocco with the international timescale would greatly benefit from additional radiometric constraints.

There is a well-established trilobite biostratigraphy for the Cambrian of southern Morocco^17^,^18^ within which the Tatelt Formation spans the *Sectigena, Hupeolenus* and *Morocconus notabilis* Biozones (Fig. 3). Unfortunately, this biostratigraphy is not well constrained by radiometric ages or chemostratigraphy, and a high degree of trilobite endemism has hindered correlation beyond the Iberian Peninsula and, to some extent, Avalonia^19^,^20^. A single radiometric age from the upper Lemdad Formation, *Antatlasia guttapluviae* Zone (Fig. 3), of 515.56 ± 1.16 Ma^21^ (recalculated from 517.0 ± 1.5 Ma^22^) provides a lower age boundary in this section. However, the Tatelt Formation is generally considered to straddle the lower – middle Cambrian (Series 2–3) boundary in Morocco, with the upper part deposited in Stage 5^15^,^16^,^20^.

The specimen was recovered from a medium-bedded well-indurated fine sand- to siltstone unit with simple trace fossils near the top of the Tatelt Formation in the Lemdad Syncline (Fig. 2B), in the *Morocconus notabilis* Zone (Fig. 3). This has been correlated to near the base of Cambrian Stage 5, possibly contemporaneous with Iberian Bilbilian/Leonian boundary and the *Lapworthella* Limestone (Ad) of the British Comley Series^18^,^19^,^20^.

## Results

### Systematic Palaeontology

Artiopoda Hou and Bergström^2^ (*sensu*^23^).

#### Remarks

The new taxon can be assigned to Artiopoda based on the preserved ventral morphology^23^, notably the antenniform first appendage pair attached at either side of a strongly sclerotized ventral hypostome, followed by numerous pairs of homonomous walking legs that gradually decrease in size along the body, and the possession of hourglass-shaped sternites. In particular, hourglass-shaped sternites rule out comparisons with non-artiopodan Cambrian euarthropods – which lack sternites altogether – such as fuxianhuiids^24^,^25^, bivalved stem-group euarthropods^26^,^27^, megacheirans^28^,^29^, and marrellomorphs^30^,^31^. Although the presence of a first pair of antenniform limbs is symplesiomorphic within Deuteropoda Ortega-Hernández^32^ (i.e. upper stem-group Euarthropoda + crown-group Euarthropoda)^33^,^34^, the combination of this character with post-oral limbs that gradually decrease in size and become differentiated into a caudal region (e.g. pygidium) are exclusive to Artiopoda.

Petalopleura Hou and Bergström^2^.

Xandarellida Chen *et al*.^1^.

#### Remarks

Hou and Bergström^2^ and Ramsköld *et al*.^3^ independently proposed definitions of Xandarellida that differ somewhat in their emphasis on particular morphological characters. We follow the diagnosis provided by Ramsköld *et al*.^3^ as this is based on a more comprehensive consideration of the organization of the cephalic appendages, hypostome morphology, and presence of segmental mismatch between the trunk limbs and tergites.

The similar style of preservation observed on the limbs and the hypostome strongly suggest that the new taxon lacked a biomineralized exoskeleton, and thus rules out potential affinities with Trilobita. The fossil is recognized as a member of Xandarellida based on similarities in limb morphology and possession of a natant hypostome associated with frontal organs (see detailed discussion below).

*Xandarella* Hou *et al*.^4^.

#### Constituent taxa

*Xandarella spectaculum* Hou *et al*.^4^ Cambrian (Stage 3) Chiungchussu Formation, Chengjiang, South China (type species); *Xandarella mauretanica* sp. nov., Cambrian (Stage 5) Tatelt Formation, High Atlas, Morocco.

#### Emended diagnosis

Semicircular head shield with small sessile eyes placed laterally. Suture or unfused overlap between shield portions extending from eye to lateral margin. Natant hypostome with elongate suboval outline. Head with antennae and up to six appendage pairs under large head shield; endopod of first post-antennal appendage reduced. Each tergite on anterior half of trunk covers a single pair of biramous appendages. Each succeeding tergite on posterior half of trunk covers an increasing number of appendage pairs, ranging from two to twelve (or more). Endopods slender, with up to a dozen podomeres. Pygidium with median posterior spine.

#### Remarks

**Figure 4:**
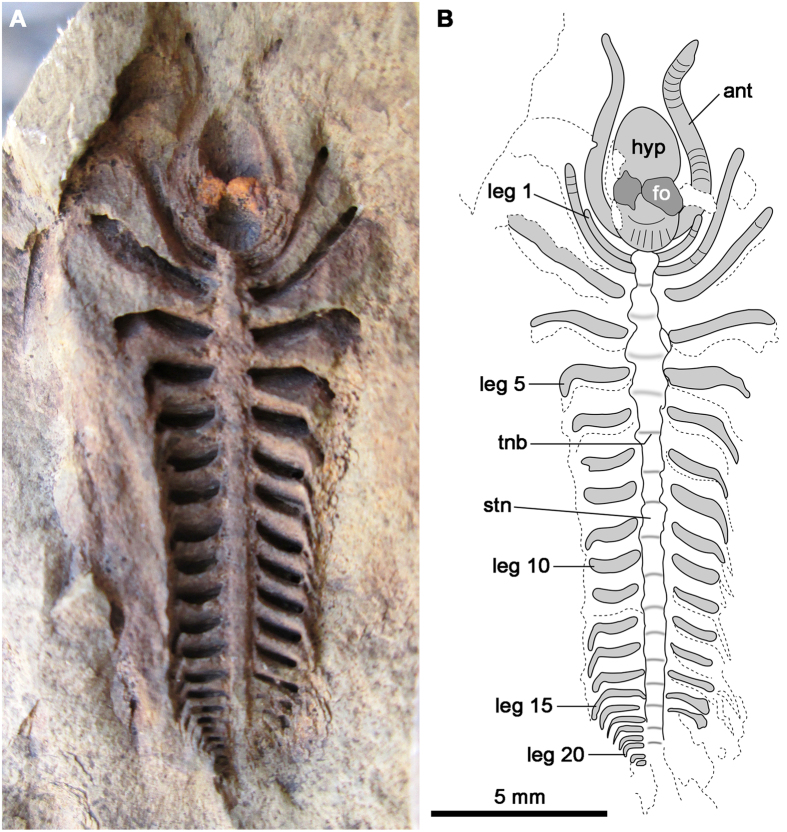
*X. mauretanica* sp. nov. from the middle Cambrian Tatelt Formation in the High Atlas of Morocco. (**A**) Holotype MHNM-HA-TT-CA-1A. (**B**) Interpretative diagram of the preserved ventral morphology. Abbreviations: *ant*, antennae; *fo*, frontal organ; *hyp*, hypostome; *stn*, sternite; *tnb*, tendinous bar.

The diagnosis of *Xandarella* has been revised from Hou *et al*.^4^ and Hou and Bergström^2^ to reflect the variability in the ventral morphology in this taxon, in light of the new specimen from Morocco (Fig. 4). The post-antennal endopods of most artiopodans possess up to seven podomeres (e.g. *Cheloniellon*^35^; *Triarthrus*^36^; *Cindarella*^3^; *Phacops* sp.^37^; *Kuamaia* and *Saperion*^5^; *Misszhouia* and *Naraoia*^38^; *Emeraldella*^23^; *Sidneyia*^39^; *Arthroaspis*^40^) as also expressed in several extant representatives, and generally resolved as the ancestral state for crown-group Euarthropoda^41^. By contrast, *X. spectaculum* and the new species described here are typified by the presence of post-antennal endopods with approximately 12 podomeres, leading us to propose this character as a diagnostic feature of *Xandarella*. A high podomere count is not exclusive to *Xandarella* among Palaeozoic euarthropods, however, as a similar condition is also known in some early Cambrian forms, such as megacheirans (e.g. *Fortiforceps*^2^), fuxianhuiids (e.g. *Fuxianhuia*^24^; *Chengjiangocaris*^25^), and bivalved stem-group euarthropods (e.g. *Jugatacaris*^42^). The substantial phylogenetic distance between these euarthropods and *Xandarella* (see topology in Legg *et al*.^43^), however, suggests that endopods with more than seven podomeres are a symplesiomorphy of Deuteropoda^29^,^33^,^34^,^41^, and its occurrence within Xandarellida is most likely a result of homoplasy. Thus, the taxonomic utility of this character for *Xandarella* is only applicable within the context of Artiopoda.

**Figure 5:**
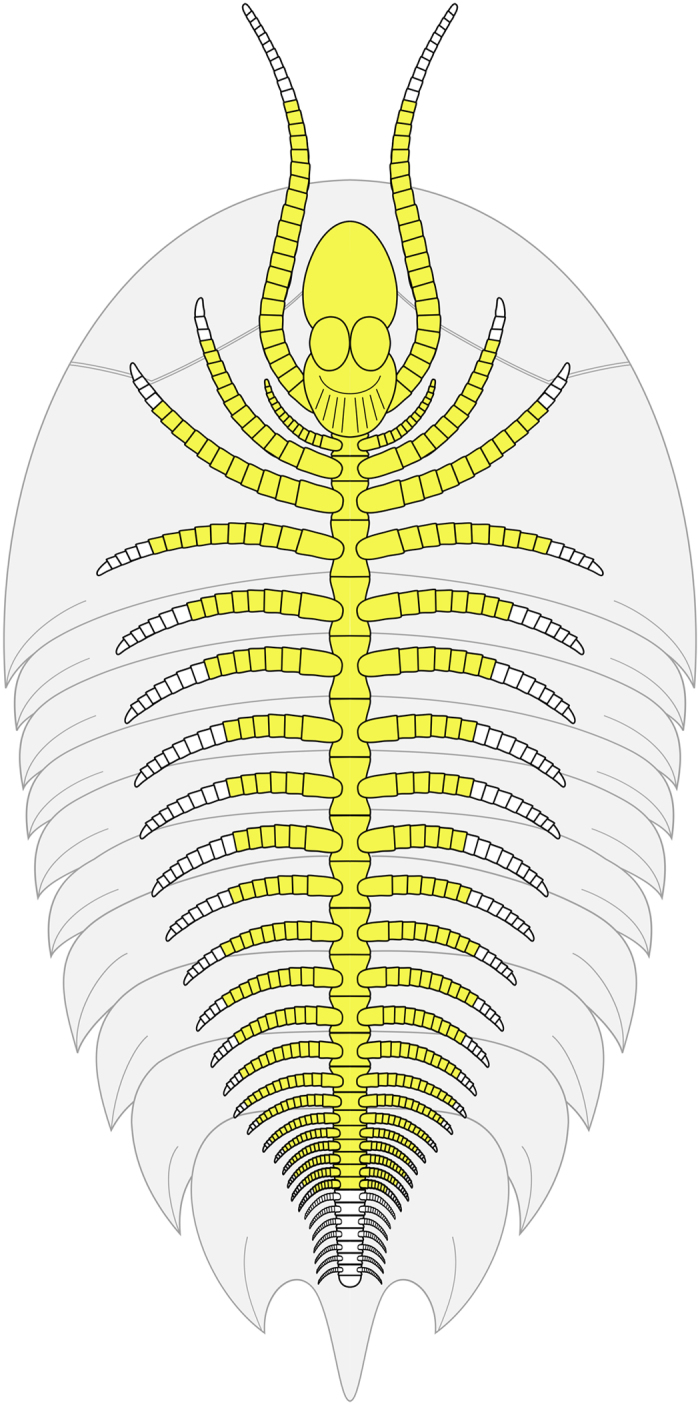
Morphological reconstruction of *Xandarella mauretanica* sp. nov. Observed ventral morphology is highlighted in yellow. Note that all aspects of the dorsal exoskeleton are hypothetical, and based on comparisons with *Xandarella spectaculum*^2^,^4^.

*Xandarella mauretanica* sp. nov. Figs 4 and 5.

#### Etymology

From the Latin *mauretanicus*, in reference to Mauretania, a historical region that corresponds to part of North Africa, including the Mediterranean coast of Morocco.

#### Diagnosis

Xandarellid with robust antennae, and a prominent hypostome with paired frontal organs located medially. At least 22 pairs of post-antennal limbs are present along the body. Endopod of first pair of post-antennal limbs half the length relative to that of the succeeding appendages.

#### Material, locality, horizon

A single specimen MHNM-HA-TT-CA-1A preserved as a ventral impression (Fig. 4), collected from the middle Cambrian (Stage 5) upper Tatelt Formation (*Morocconus notabilis* Zone) of Morocco.

#### Description

**Figure 6:**
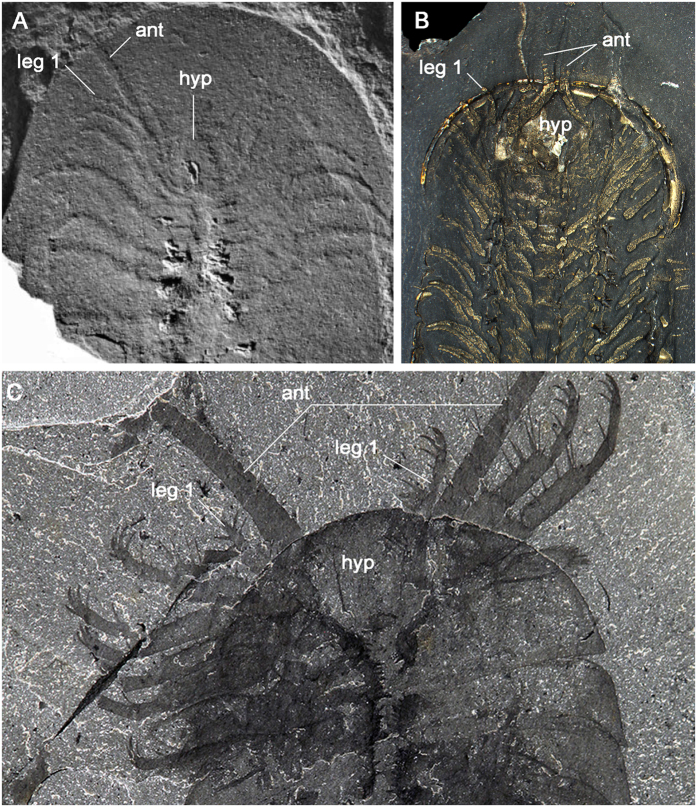
Anterior limb morphology of selected Cambrian artiopodans. (**A**) *Campanamuta mantonae*, Cambrian (Stage 3) Sirius Passet, North Greenland^50^. (**B**) *Triarthrus eatoni*, Upper Ordovician, Beecher’s Trilobite Bed, USA^36^. (**C**) *Emeraldella brocki*, ROM-61148, Cambrian (Stage 5) Burgess Shale, British Columbia^23^ (photograph courtesy of Jean-Bernard Caron, Royal Ontario Museum). Abbreviations: *ant*, antennae; *hyp*, hypostome.

The holotype is an articulated, and almost complete, individual with a total length of 21 mm (sagittal; including the antennae), and maximum width of 8 mm (transverse) (Fig. 4). The specimen represents an exceptionally preserved impression of the lightly sclerotized underside of the body, including appendages and ventral exoskeletal elements. Details of the dorsal exoskeleton, including cephalon and trunk tergite morphology, are entirely absent. The cephalic region incorporates a prominent hypostome with an elongate suboval outline (ca. 4 mm length, sag.; 1.8 mm maximum width, trans.), expressed as a deep concave impression, and typified by a medial transverse constriction that conveys an approximately lemniscate appearance. Two convex rounded structures (ca. 0.9 mm diameter) are associated with the medial constriction; these features are tentatively interpreted as a pair of frontal organs, simple ocelli-like structures found on the anterior region of various artiopodans^5^,^44^. The frontal organs superficially bisect the hypostome. The anterior half has a slightly acute anterior margin and a smooth texture. The posterior half has a rounded posterior margin, and evinces ornamentation consisting of a single transverse and broad, crescentic ridge located medially, followed posteriorly by six longitudinal ridges arranged in a parallel series. The suboval outline of the hypostome indicates a natant attachment to the underside of the head, as concomitant hypostomes invariably possess a blunt anterior edge that matches the cephalic margin, or extends from the cephalic doublure (examples discussed below) (Fig. 6B,C).

A pair of multiannulated antennae attach at either side of the hypostome, immediately posterior to the position of the frontal organs. The antennae are both longer (5 mm, sag.) and wider (0.75 mm, trans.) than any of the other preserved appendages, and demonstrate a distinctive sigmoidal flexure towards the anterior end of the body. Fine morphological details are mostly indistinct, but faint segmental boundaries (ca. 0.25 mm length, sag.) suggest that the antennae are composed of at least 20 podomeres or annuli; the length of the annuli is consistently shorter than their width along the preserved extent of the antenna. The holotype preserves a total of 22 pairs of slender concave impressions of variable length, which correspond to the endopods of the post-antennal appendages. The 1^st^ pair of post-antennal appendages originates in close proximity to the posterior margin of the hypostome, and curves anteriorly until reaching the same level as the posterior border of the frontal organs. These delicate appendages are both shorter (ca. 1.3 mm, sag.) and thinner (0.25 mm, trans.) than any other pair on the anterior two thirds of the body. The 2^nd^ and 3^rd^ leg pairs share approximately the same dimensions (ca. 3.5 mm length, sag.; 0.6 mm width, trans.) and are similarly curved anteriad. Faint segmental impressions (ca. 0.25 mm length, sag.) on the distal end of the 2^nd^ leg indicate podomeres that are of subequal length and width, or slightly longer than wide; similar impressions more proximally also indicate podomeres that are slightly longer than wide, suggesting the presence of up to a dozen podomeres per limb, at least on the cephalic region. The 4^th^ leg pair is slightly shorter than the preceding one (ca. 3 mm length, sag.), and is laterally splayed rather than flexed anteriorly. Unlike the 2^nd^ and 3^rd^ legs, the 4^th^ pair evinces a distal curvature towards the posterior end, which becomes more accentuated in the subsequent appendages. The subtle decrease in size and shift in appendage orientation suggest that the 4^th^ leg pair could correspond to the last set of cephalic appendages; if correct, this interpretation would imply that the head includes the antennae and four pairs of (arguably) biramous appendages. This interpretation may be supported by the fact that the 2^nd^ to 4^th^ legs display a regular separation of 1 mm (sag.), whereas the spacing between the succeeding pairs progressively decreases towards the posterior end of the specimen. The 5^th^ to 15^th^ legs have the same overall construction, consisting of a laterally oriented concave impression with a small posterior curvature at the distal end, and only differ slightly in their dimensions. Although the 5^th^ leg has an approximate length of 2.4 mm (sag.) and width of 0.6 mm (trans.), the same measurements are 1.6 mm (sag.) and 0.4 mm (trans.) for the 15^th^ leg, reflecting a gentle decrease in overall size towards the posterior end. This organization gives the appearance of a narrowed trunk relative to the anterior cephalic region, although this is mostly applicable to the proximal parts of the limbs, and thus there is no reason to assume that the dorsal exoskeleton would necessarily follow this morphology. The 16^th^ to 22^nd^ legs show a more drastic decrease in size resulting in a triangular tapering of the body; although the 16^th^ leg is only slightly shorter than the preceding limb (ca. 1.3 mm, sag.), the 22^nd^ leg is only expressed as a suboval impression of approximately 0.15 mm in length (sag.). This sharp change in appendage length may reflect the fusion of posterior segments into a discrete pygidium. The caudal termination of the trunk is not observed.

The only exoskeletal elements preserved on the ventral side – other than the hypostome – correspond to the sternite (i.e. ventral sclerotized plate) series, which occupies a longitudinal axial position between the limb pairs. Unlike the appendages, the sternite series is differentially preserved as a convex impression that reflects the pattern of segmentation through an alternating series of light and dark bands of sediment. The light bars are aligned with the appendage impressions and generally possess a narrower profile (trans.) relative to the dark bands; this disposition indicates that the bands represent the sternites and tendinous bars (i.e. intersegmental arthrodial membrane) respectively. The length of the individual sternites mirrors the spacing of the appendage pairs along the body, and thus the longest (sag.) are located between the 3^rd^ and 5^th^ legs, and become progressively shorter towards the rear termination of the body. Despite the gradual reduction in appendage size, the width of the sternite series remains relatively invariant throughout the length of the body – as informed by the separation between the leg impressions – with a maximum and minimum width (trans.) of 1 mm (at the 4^th^ leg) and 0.8 mm (at the 15^th^ leg) respectively.

#### Remarks

*X. mauretanica* sp. nov. differs from *X. spectaculum* in that the 1^st^ leg pair in the former is distinctively reduced (Figs 4 and 5), whereas in the latter species these appendages follow a more gentle gradation in size with the succeeding cephalic legs^2^,^45^. *X. spectaculum* also differs in featuring up to 36 pairs of post-antennal leg pairs^2^, compared to the 22 leg pairs preserved in the new taxon. Whether this difference may be attributed to the preservation of *X. mauretanica* sp. nov., or if it reflects actual interspecific variability, remains uncertain. It is also possible that the different number of post-antennal legs can be attributed to ontogeny, as the holotype *X. mauretanica* sp. nov. is significantly smaller (length 21 mm, sag.) than type material of *X. spectaculum* (e.g. holotype, length 51 mm length, sag.^2^). Clarification on these issues will require the input of additional material of *X. mauretanica* sp. nov., or studies on the ontogeny of *X. spectaculum*.

## Discussion

### Phylogenetic affinities

Although only details of the ventral anatomy are preserved in the available material, the similarities between *X. mauretanica* sp. nov. and *X. spectaculum* support their close phylogenetic affinities, and offers new insights on the morphological variability within Xandarellida. The new taxon confirms the presence of hourglass-shaped sternites connected by intersegmental tendinous bars – previously suggested^2^ or inferred^3^ for members of Xandarellida – similarly to the ventral exoskeletal anatomy of other artiopodans^36^,^38^,^46^,^47^. The appendicular organization in *X. mauretanica* sp. nov. and *X. spectacullum* share various symplesiomorphies of Artiopoda, most notably the presence of antennae at either side of a sclerotized hypostome, the homonomous construction of the post-oral appendages, and the progressive reduction in size of the legs towards the posterior end of the body. However, the presence of post-antennal endopods with approximately 12 podomeres is unique to *Xandarella* within the evolutionary context of Artiopoda^48^.

These comparisons are further strengthened by the presence of a natant hypostome in *X. mauretanica* sp. nov. and *X. spectaculum*. Several Cambrian artiopodans possess a concomitant hypostome that is widely attached to the anterior margin of the cephalon, and may be expressed as an extension of the cephalic doublure with or without a suture (e.g. *Triarthrus*^36^; *Emeraldella*^23^; *Squamacula*^49^; *Aglaspis*^48^) (Fig. 6B,C) or occur in association with an anterior sclerite (e.g. Conciliterga^2^,^44^; see also char. 12 in Edgecombe and Ramsköld^5^). By contrast, the natant hypostome of Xandarellida is situated further back, in a position closer to the sagittal midline of the head^2^,^3^. Although the natant hypostome is also known in various non-trilobite artiopodans (e.g. *Cheloniellon*^35^; Nektaspida^10^,^38^; *Campanamuta*^50^; *Arthroaspis*^40^) (Fig. 6A), none of these taxa combine this character with the presence of endopods with more than seven podomeres as observed in *Xandarella*.

The ventral anatomy of *X. mauretanica* sp. nov. is broadly comparable to that of *Cindarella eucalla* in terms of overall appendage organization. However, these taxa differ in that the 1^st^ leg pair of the latter is not reduced^3^, and the hypostome of the former is more elongate and bears the paired frontal organs (Figs 4 and 5); the endopods of *C. eucalla* also differ in evidently having archetypal endopods with seven podomeres^3^. Comparisons with the recently described xandarellid *Luohuilinella rarus* are problematic because this taxon is only known from the dorsal exoskeleton^6^. However, the posterior end of the body in *X. mauretanica* sp. nov. and *L. rarus* exhibits a sharp decrease in width that produces a distinctive subtriangular caudal tapering, which is otherwise not observed in either *X. spectaculum* or *C. eucalla*^2^,^3^, or other non-trilobite artiopodan groups (e.g. Cheloniellida^35^; Conciliterga^51^; Nektaspida^38^; Xenopoda^23^).

Outside the Xandarellida, the morphology of *X. mauretanica* sp. nov. shares similarities with the petalopleuran *Sinoburius lunaris*^4^. With the exception of the 1^st^ leg pair in *X. mauretanica* sp. nov., the cephalic appendages of both taxa are noticeably longer than those in the trunk region; however, the possibility that this appearance in the new taxon may be a taphonomic artefact cannot be entirely discarded at present given the preferential preservation of the proximal portion of the appendages (Fig. 4). *S. lunaris* may further resemble *X. mauretanica* sp. nov., in the possession of four legs in the head region, yet again, assuming that the extrapolation of the cephalic shield based on the anatomy of the anterior appendages is correct. The preservation of *S. lunaris* only reveals the outline of the endopods, and thus the number of constituent podomeres is uncertain. Less cryptically, the paired frontal organs in the hypostome of *X. mauretanica* sp. nov. draw a parallel to similar structures in *S. lunaris*^2^,^5^, although the natant hypostome in the latter taxon is comparatively smaller and has a subtriangular outline. The frontal organs of *S. lunaris* appear to be located in a slightly anterior position relative to the hypostome, however, and thus it is uncertain if they reflect an identical organization to that observed in *X. mauretanica* sp. nov. The pygidial segmentation of *S. lunaris* also displays a sharp decrease in limb size, comparable to that of *X. mauretanica* sp. nov. (Fig. 4) and *L. rarus*^6^.

### Taphonomic implications

The preservation of *X. mauretanica* sp. nov. is noteworthy in comparison to other xandarellid specimens, and indeed to other soft-bodied Cambrian euarthropods. Previously described xandarellid fossils from the Chengjiang biota are expressed as pyritized carbonaceous compressions in shale, as is typical for non-trilobite euarthropods^52^. The holotype of *X. mauretanica* sp. nov. is three-dimensionally preserved in a fine sand- to siltstone (Fig. 4) and was probably pyritized during early diagenesis, which would account for the exceptional preservation of the lightly sclerotized ventral morphology; however, the appearance of the fossil strongly suggests a more recent exposure to oxidation and intense weathering. This peculiar style of three-dimensional preservation contrasts with that of Burgess Shale-type Cambrian deposits in Laurentia, consisting of flattened carbonaceous films^53^, and also with the non-biomineralized euarthropods from the Early Ordovician Fezouata biota of Morocco^54^. Thus, the taphonomy of the fossils at the Tatelt Formation requires further investigation, but demonstrates that soft-bodied Burgess Shale-type euarthropods can be found in atypical sedimentological settings.

### Stratigraphic and palaeobiogeographic significance

**Figure 7:**
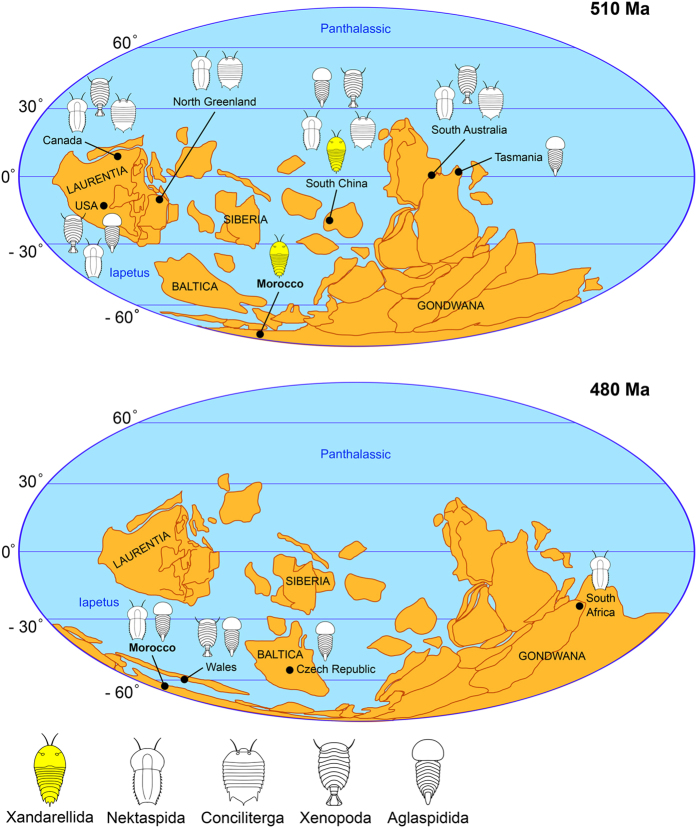
Palaeobiogeographical distribution of major groups of Lower Palaeozoic Artiopoda during the Cambrian and Ordovician. *Xandarella mauretanica* sp. nov. represents the only member of Xandarellida reported outside South China, and expands the distribution of this clade to the South Hermisphere. References for Cambrian localities: Laurentia^23^,^39^,^51^,^58^,^59^,^60^,^61^; North Greenland^40^,^62^; Morocco (this study); South China^2^,^63^,^64^,^65^; South Australia^9^,^10^,^66^; Tasmania^67^. References for Ordovician localities: Baltica^68^; Wales^69^,^70^; Morocco^54^,^56^,^71^; South Africa^72^. Palaeocontinental reconstructions redrawn, adapted and simplified by J.O.-H. from Torsvik and Cocks^73^ (Figs 2.8, 2.11) using Adobe Illustrator CC 2015.3 (http://www.adobe.com/uk/products/illustrator.html).

The discovery of *X. mauretanica* sp. nov. from the middle Cambrian Tatelt Formation in the High Atlas Mountains of Morocco provides the youngest known record of xandarellids, extending their stratigraphic range by approximately 10 million years. More significantly, this finding substantially expands the palaeobiogeographic and palaeolatitudinal range of Xandarellida out of tropical South China (Chengjiang biota) and onto polar Gondwana at high southern latitudes during the Cambrian (Fig. 7). Given that xandarellids are not known from the early Cambrian (Stage 4) Emu Bay Shale in South Australia^11^,^55^, *X. mauretanica* sp. nov. represents the only direct connection between communities of non-biomineralized artiopodans in continental Gondwana (North Africa) and South China (Chengjiang biota) during the Cambrian (Fig. 7). The close palaeobiogeographical links between Morocco and South China only become evident later on during the latest Tremadocian (Lower Ordovician) thanks to the euarthropod diversity preserved in the Fezouata biota, which includes representatives of several typically Cambrian groups such as marrellomorphs, leanchoiliids, mollisoniids, nektaspids and aglaspidids^54^,^56^,^57^ (Table 2). This raises the possibility that Burgess Shale-type euarthropod communities in Gondwana are not necessarily restricted to the Emu Bay Shale in South Australia, but that they may also extend to the early Cambrian of Morocco.

The discovery of *X. mauretanica* sp. nov. draws attention to the absence of xandarellids from several Cambrian Burgess Shale-type faunas in North America (Fig. 7) (Table 2). Given the intense efforts invested in the systematic description of non-biomineralized Cambrian euarthropods from Laurentia over the last 50 years, the absence of xandarellids from this palaeocontinent may reflect a real palaeobiogeographic signal, rather than an artefact of taphonomic or collection bias. Future work on the Tatelt Formation offers great potential for the discovery of additional exceptionally preserved fossils in the middle Cambrian of Morocco that will help further refine the palaeobiogeographic and stratigraphic distribution of Burgess Shale-type faunas.

## Materials and Methods

A single available specimen collected from the upper Tatelt Formation (*Morocconus notabilis* Zone) of Morocco. Specimen MHNM-HA-TT-CA-1A corresponds to the external mould of the ventral side of the body in dorsoventral view, preserved in a medium-bedded well-indurated mudstone and sandstone unit. Photographs were taken with a Nikon 3100 DSLR. The material is housed at the MHNM (Natural Museum History of Marrakesh).

## Additional Information

**How to cite this article**: Ortega-Hernández, J. *et al*. A xandarellid artiopodan from Morocco – a middle Cambrian link between soft-bodied euarthropod communities in North Africa and South China. *Sci. Rep.* **7**, 42616; doi: 10.1038/srep42616 (2017).

**Publisher's note:** Springer Nature remains neutral with regard to jurisdictional claims in published maps and institutional affiliations.

## Figures and Tables

**Figure 1:**
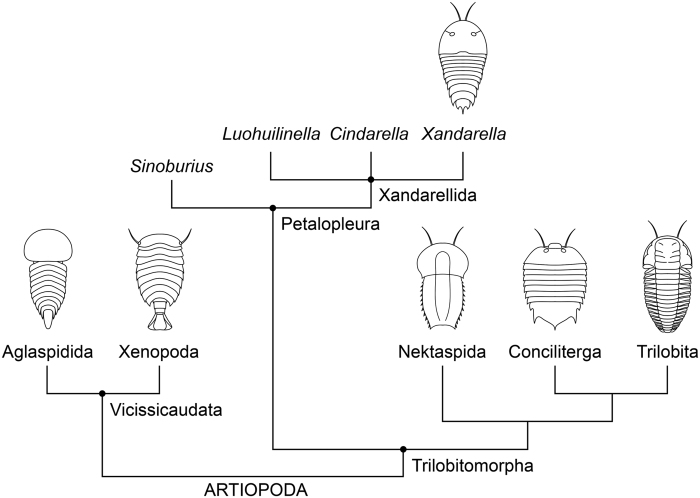
Simplified phylogeny of Artiopoda. Topology follows Ortega-Hernández *et al*.^48^.

**Table 1:** Diversity of Cambrian Petalopleura Hou and Bergström^2^.

Taxon	Classification	Age	Locality	References
Cindarella eucalla*	Xandarellida	Cambrian Stage 3	Chiungchussu Fm., Chengjiang, South China	Chen *et al*.^1^
				Ramsköld *et al*.^3^
				Hou and Bergström^2^
*Xandarella spectaculum*	Xandarellida	Cambrian Stage 3	Chiungchussu Fm., Chengjiang, South China	Hou *et al*.^4^
				Ramsköld *et al*.^3^
				Hou and Bergström^2^
*Luohuilinella rarus*	Xandarellida	Cambrian Stage 3	Chiungchussu Fm., Chengjiang, South China	Zhang *et al*.^6^
*Sinoburius lunaris*	Sinoburiida	Cambrian Stage 3	Chiungchussu Fm., Chengjiang, South China	Hou *et al*.^4^
				Hou and Bergström^2^
*Phytophilaspis pergamena*	Unranked	Cambrian Stage 4	Sinsk Fm., Sinsk, Siberia	Ivanstov^8^
*Xandarella mauretanica*	Xandarellida	Cambrian Stage 5	Tatelt Formation, Morocco	This study

*Note that the putative xandarellid *Almenia spinosa*^2^ has been regarded as a synonym of *Cindarella eucalla* (see Edgecombe and Ramsköld^5^).

**Table 2:** Comparison of palaeobiogeographic and stratigraphic occurrence of major groups of Burgess Shale-type non-trilobite euarthropods in Gondwana and Laurentia.

Taxon	Morocco		China	Australia	North America
	Cambrian	Ordovician	Cambrian	Cambrian	Cambrian
Aglaspidida	none	Van Roy *et al*.^56^	Lerosey-Aubril *et al*.^64^	none	Hesselbo^74^
		Ortega-Hernández *et al*.^71^			Lerosey-Aubril *et al*.^61^
Bivalved stem euarthropods	none	Van Roy *et al*.^54^	Yang *et al*.^75^Fu and Zhang^42^	García-Bellido *et al*.^26^	Briggs^76^Legg and Caron^27^
Conciliterga	none	none	Hou and Bergström^2^	Paterson *et al*.^9^	Whittington^51^Ortega-Hernández^44^
Marrellomorpha	none	Van Roy *et al*.^56^Legg^77^	Liu^78^	none	García-Bellido and Collins^30^
Megacheira	none	Van Roy *et al*.^54^	Chen *et al*.^79^Liu *et al*.^80^	Edgecombe *et al*.^81^Paterson *et al*.^55^	Haug *et al*.^28^Aria *et al*.^29^
Mollisoniida	none	Van Roy *et al*.^56^	Zhang *et al*.^65^	none	Walcott^82^
Nektaspida	none	Van Roy *et al*.^54^	Zhang *et al*.^38^	Paterson *et al*.^10^	Walcott^82^Whittington^58^
Xandarellida	**this study**	none	Ramsköld *et al*.^3^Zhang *et al*.^6^	none	none
Xenopoda	none	none	Zhang *et al*.^63^	Edgecombe *et al*.^66^	Stein and Selden^23^Stein^39^

Note that the list is not exhaustive, but rather serves as a broad comparison between non-trilobite euarthropod communities in these regions.
