# Corrigendum: A xandarellid artiopodan from Morocco – a middle Cambrian link between soft-bodied euarthropod communities in North Africa and South China

**DOI:** 10.1038/srep46797

**Published:** 2017-05-09

**Authors:** Javier Ortega-Hernández, Abdelfattah Azizi, Thomas W. Hearing, Thomas H. P. Harvey, Gregory D. Edgecombe, Ahmid Hafid, Khadija El Hariri

Scientific Reports 7: Article number: 4261610.1038/srep42616; published online: 02 17 2017; updated: 05 09 2017

This Article contains errors in Figure 7. The correct Figure 7 appears below as Figure 1 with a corrected Figure legend and additional references:

## Figures and Tables

**Figure 1:**
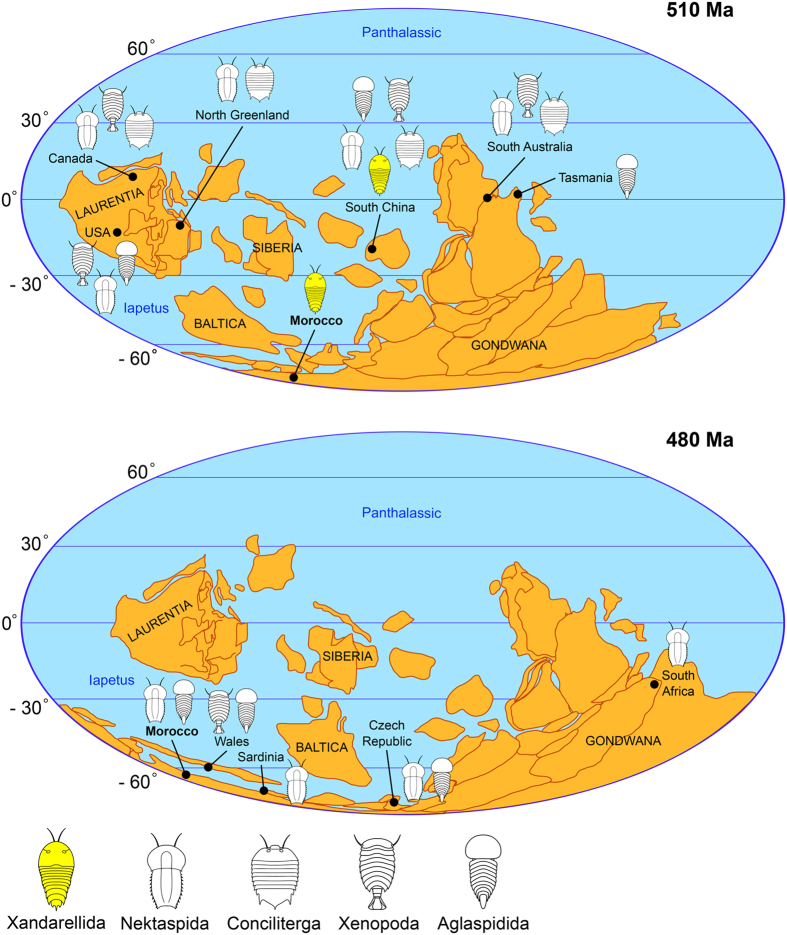
Palaeobiogeographical distribution of major groups of Lower Palaeozoic Artiopoda during the Cambrian and Ordovician. *Xandarella mauretanica* sp. nov. represents the only member of Xandarellida reported outside South China, and expands the distribution of this clade to the Southern Hemisphere. References for Cambrian localities: Laurentia^23,39,51,58–61^; North Greenland^40,62;^ Morocco (this study); South China^2,63–65^; South Australia^9,10,66^; Tasmania^67^. References for Ordovician localities: Wales^69,70^; Morocco^54,56,71^; South Africa^72^; Sardinia^1^; Czech Republic^2^,^68^. Palaeocontinental reconstructions based on Torsvik and Cocks^73^ (Fig. 2.8, 2.11) using Adobe Illustrator CC 2015.3 (http://www.adobe.com/uk/products/illustrator.html).
